# Supplementary material for: Fibroblast mediated dynamics in diffusively uncoupled myocytes: a simulation study using 2-cell motifs
Source: Sci Rep. 2024 Feb 24;14:4493. doi: 10.1038/s41598-024-54564-1 (PMC10891142; doi:10.1038/s41598-024-54564-1)
Supplement: Supplementary file 1 — Supplementary Figures. [file 41598_2024_54564_MOESM1_ESM.pdf]

SUPPLEMENTARY MATERIAL

Fibroblast mediated dynamics in diffusively uncoupled myocytes - a simulation study using 2-cell motifs.

S. Sridhar and R. H. Clayton

Contents

1. Fig S1: 2-parameter plots for all the 3 motifs describing the effect of the coupling strengths G_{Loc}, G_{Long} on APD for both $myocyte - 1$ (a,c,e) and $myocyte - 2$ (b,d,f) with *Shallow* parameters.

2. Fig S2: Action potential profiles for both myocytes in $Motif - 1$ while pacing the cells at $T = 600$ ms at $G_{Loc} = G_{Long} = 0.5$ nS (a), 2.0 nS (b) and 4.0 nS (c) respectively. The blue and red traces correspond to *Shallow* and *Steep* parameter sets.

3. Fig S3: Action potential profiles for both myocytes in $Motif - 3$ while pacing the cells at $T = 600$ ms at $G_{Loc} = G_{Long} = 0.5$ nS (a), 2.0 nS (b) and 4.0 nS (c) respectively. The blue and red traces correspond to *Shallow* and *Steep* parameter sets.

4. Fig S4: Effect of delay in stimulation of $myocyte - 2$ on the difference in the APD of the two myocytes for the different motifs with *Shallow* parameters. The values of the delay are $\tau_D = 0$ ms (a,d,g), $= 10$ ms (b, e, h) and $= 25$ ms (c, f, i).

5. Fig S5: The time series of trans-membrane potential for both the myocytes in $Motif - 3$, with $myocyte - 2$ stimulated 25 ms after $myocyte - 1$.

6. Fig S6: 2-parameter conductance map for pacing period $T = 600$ ms describing the different dynamical regimes for the *Steep* parameter set, *viz.,* NR, IR and $1:1$, characterised as the fraction of stimuli that elicit an action potential in the *Response Cell* for $Motif - 1$ (a,b) and $Motif - 2$ (c-f).

7. Fig S7: 2-parameter conductance map for pacing period $T = 600$ ms describing the different dynamical regimes for the *Shallow* parameter set, *viz.,* NR, IR and $1:1$, characterised as the fraction of stimuli that elicit an action potential in the *Response Cell* for $Motif - 1$ (a,b) and $Motif - 2$ (c-f).

8. Fig S8: 2-parameter conductance map describing the different dynamical regimes for the *Shallow* parameter, *viz.,* NR, IR and $1:1$, characterised as the fraction of stimuli that elicit an action potential in the *Response Cell* for $Motif - 2$ at pacing period $T = 300$ ms.

9. Fig S9: 2-parameter conductance map describing the different dynamical regimes for the *Steep* parameter set, *viz.,* NR, IR and $1:1$, characterised as the fraction of stimuli that elicit an action potential in the *Response Cell* for $Motif-2$ at pacing period $T = 300$ ms.

10. Fig S10: 2-parameter conductance map describing the different dynamical regimes for the *Shallow* parameter, *viz.,* NR, IR and $1:1$, characterised as the fraction of stimuli that elicit an action potential in the *Response Cell* for $Motif-1$ (a,b) and $Motif-3$ (c,d) at pacing period $T = 300$ ms.

11. Fig S11: 2-parameter conductance map describing the different dynamical regimes for the *Steep* parameter set, *viz.,* NR, IR and $1:1$, characterised as the fraction of stimuli that elicit an action potential in the *Response Cell* for $Motif-1$ (a,b) and $Motif-3$ (c,d) at pacing period $T = 300$ ms.

12. Fig S12: Effect of coupling motifs on APD for *Steep* parameters with $V_{FR} = -49$ mV.

13. Fig S13: Identifying regimes for *Steep* parameters at $T = 600$ ms that initiate action potentials in non-stimulated cells for $V_{FR} = -49$ mV.

14. Fig S14: Effect of coupling motifs with *Shallow* cell parameters on APD for $V_{FR} = -49$ mV.

15. Fig S15: Identifying conductance parameters that initiate action potentials in non-stimulated myocyte with *Shallow* restitution for $V_{FR} = -49$ mV.

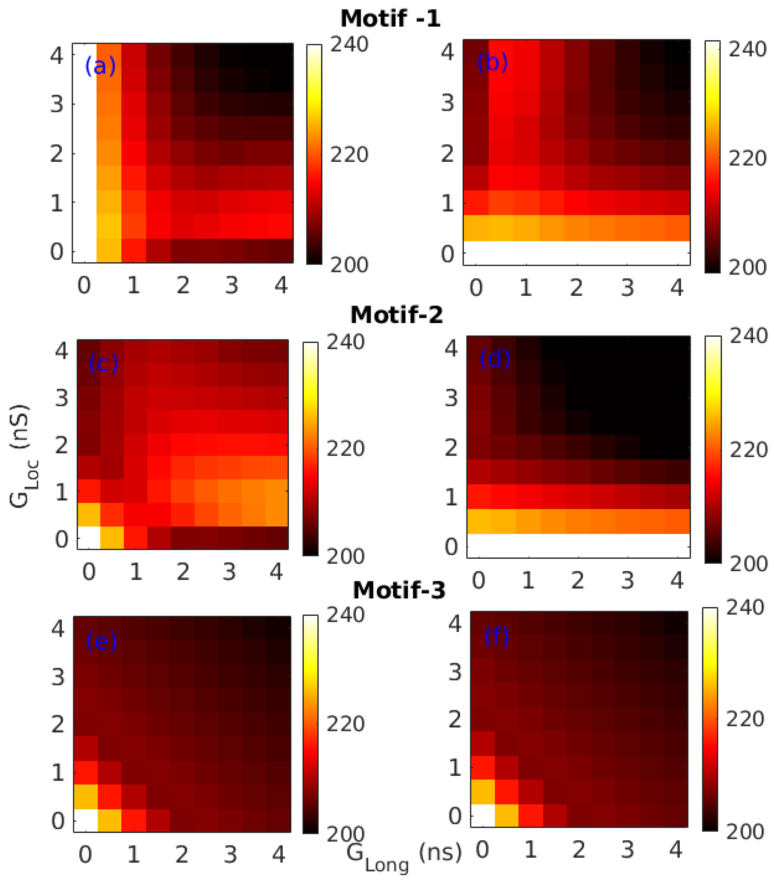

Figure 1: 2-parameter plots for all the 3 motifs describing the effect of the coupling strengths G_{Loc}, G_{Long} on the APD for both $myocyte-1$ (a,c,e) and $myocyte-2$ (b,d,f) with *Shallow* parameters.

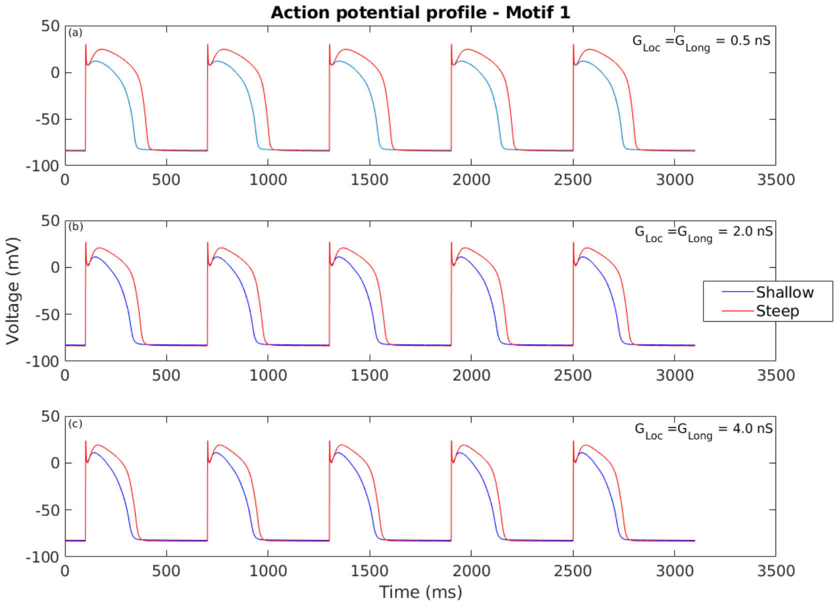

Figure 2: Action potential profiles for both myocytes in $Motif - 1$ while pacing the cells at $T = 600$ ms at $G_{Loc} = G_{Long} = 0.5$ nS (a), 2.0 nS (b) and 4.0 nS (c) respectively. The blue and red traces correspond to *Shallow* and *Steep* parameter sets.

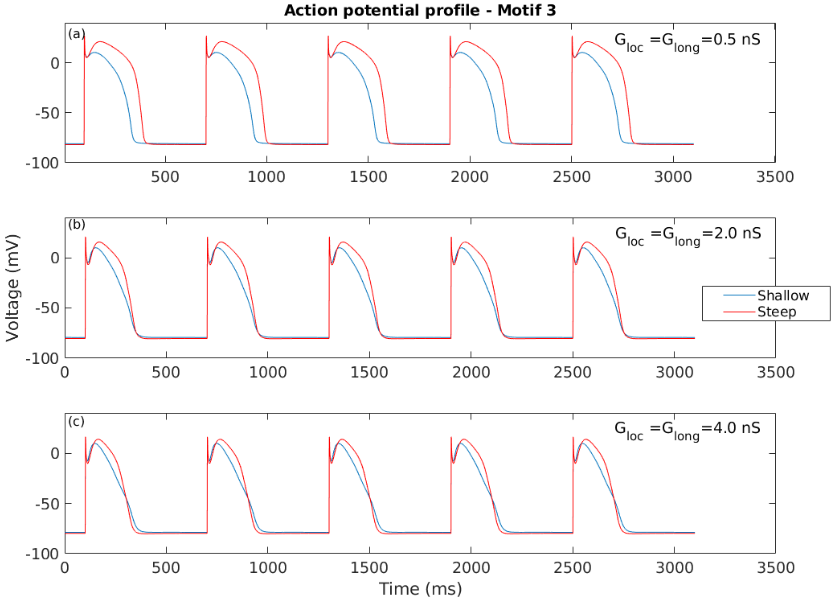

Figure 3: Action potential profiles for both myocytes in $Motif-3$ while pacing the cells at $T = 600$ ms at $G_{Loc} = G_{Long} = 0.5$ nS (a), 2.0 nS (b) and 4.0 nS (c) respectively. The blue and red traces correspond to *Shallow* and *Steep* parameter sets.

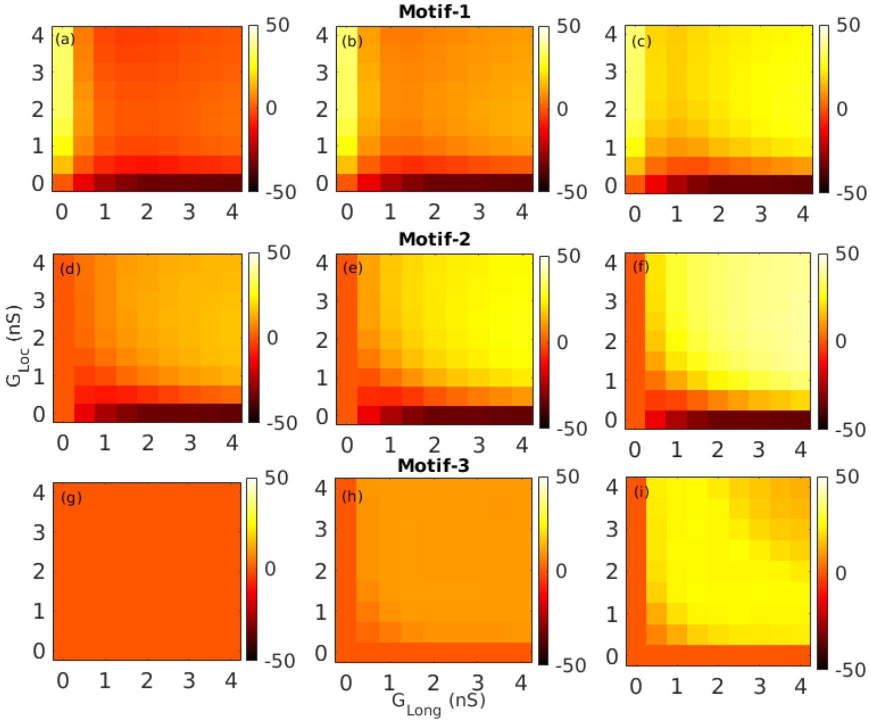

Figure 4: Effect of delay in stimulation of $myocyte - 2$ on the difference in the APD of the two myocytes for the different motifs with *Shallow* parameters. The values of the delay are $\tau_D = 0$ ms (a, d, g), $= 10$ ms (b, e, h) and $= 25$ ms (c, f, i).

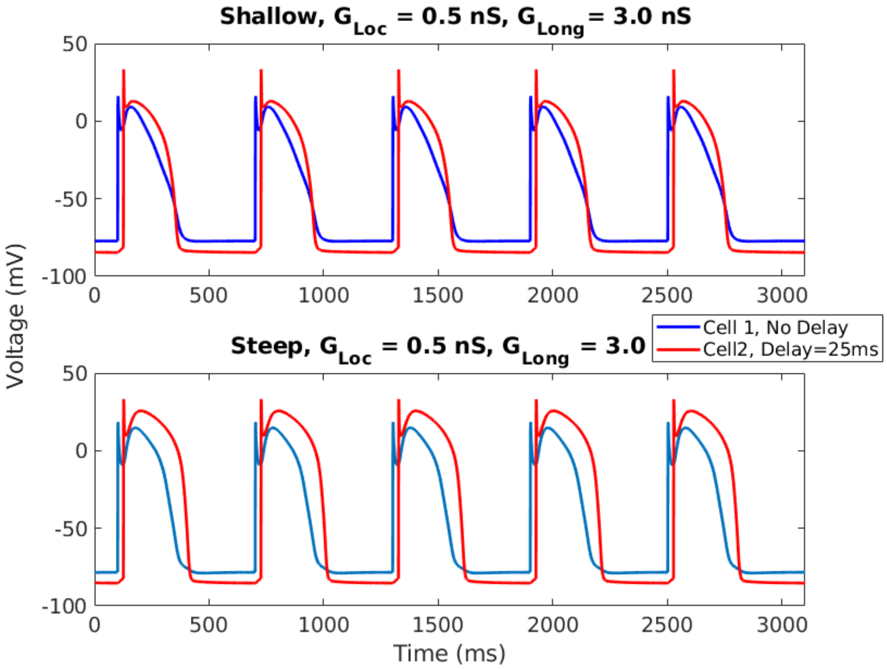

Figure 5: The time series of trans-membrane potential for both myocytes in $Motif-3$, with $myocyte-2$ stimulated 25 ms after $myocyte-1$.

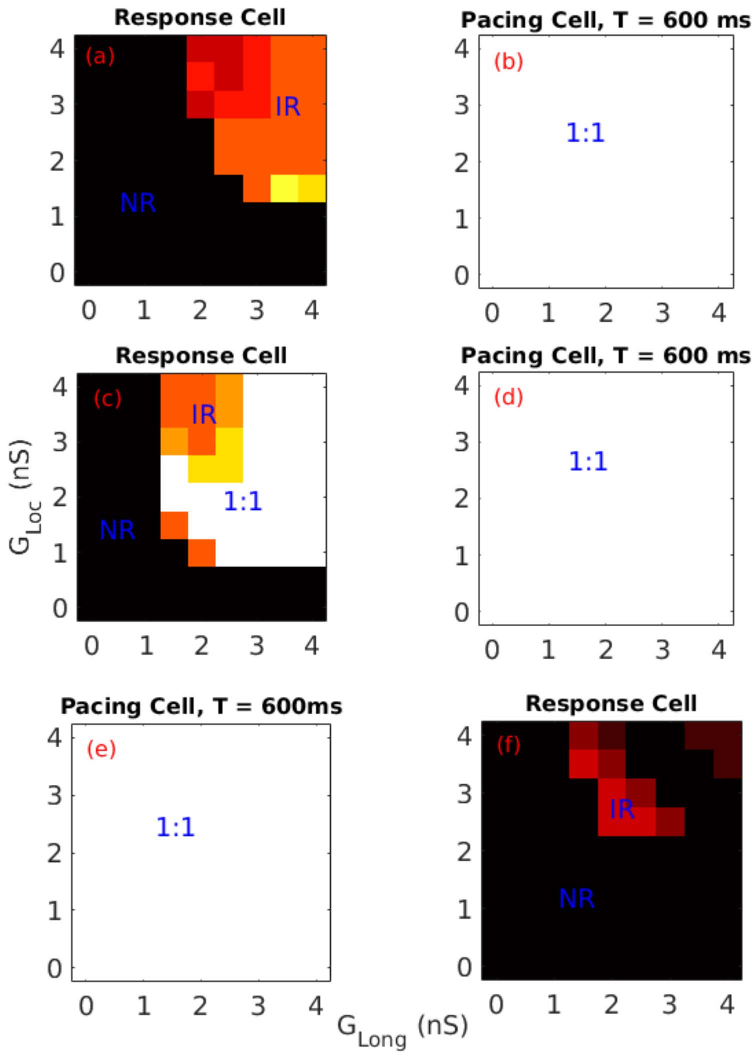

Figure 6: 2-parameter conductance map for pacing period $T = 600$ ms describing the different dynamical regimes for the *Steep* parameter set, *viz.*, NR, IR and $1:1$, characterised as the fraction of stimuli that elicit an action potential in the *Response Cell* for $Motif-1$ (a,b) and $Motif-2$ (c-f).

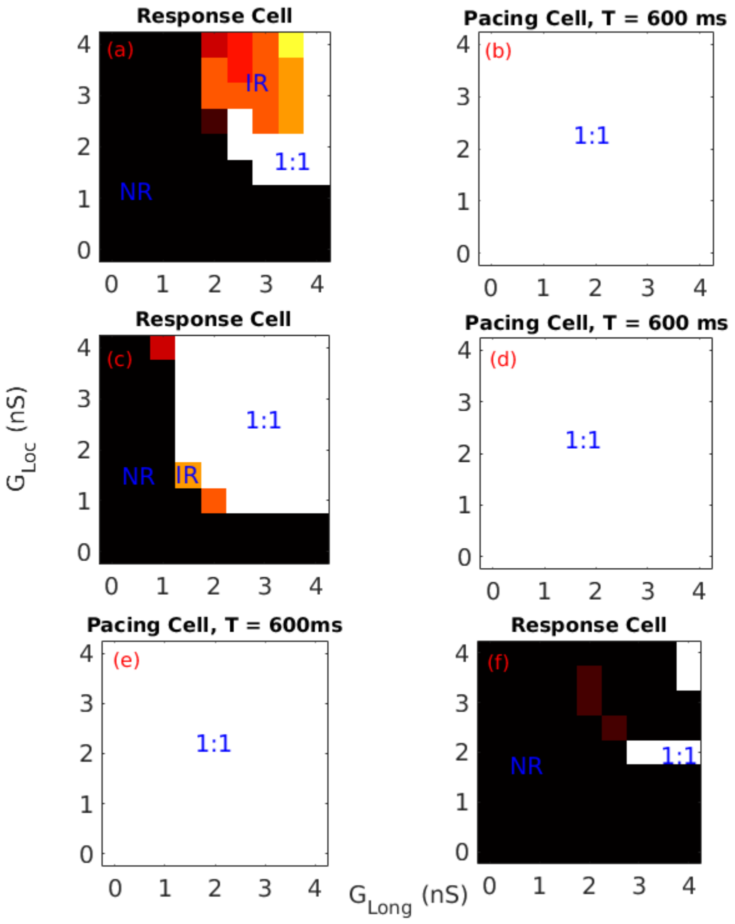

Figure 7: 2-parameter conductance map for pacing period $T = 600$ ms describing the different dynamical regimes for the *Shallow* parameter set, *viz.*, NR, IR and $1:1$, characterised as the fraction of stimuli that elicit an action potential in the *Response Cell* for $Motif-1$ (a,b) and $Motif-2$ (c-f).

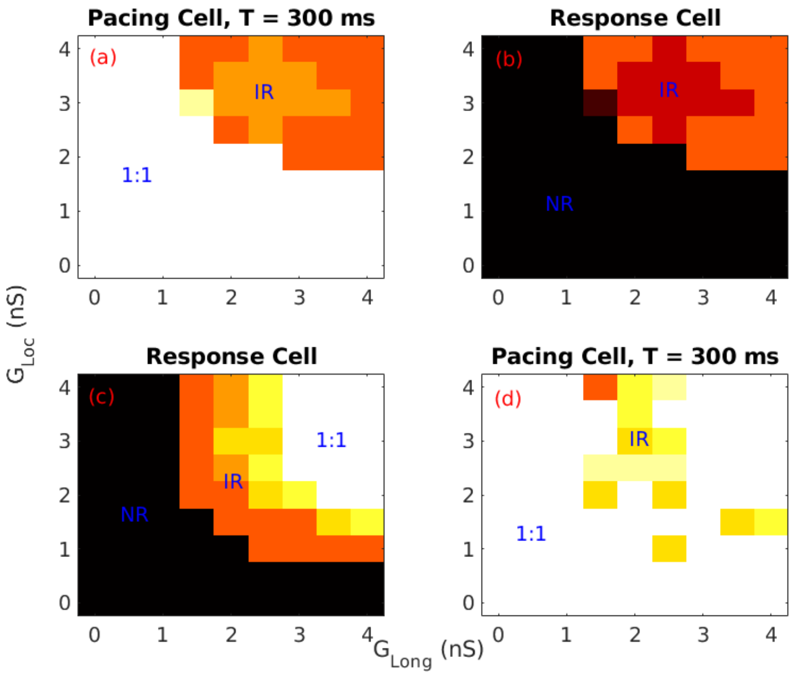

Figure 8: 2-parameter conductance map describing the different dynamical regimes for the *Shallow* parameter, *viz.*, NR, IR and $1:1$, characterised as the fraction of stimuli that elicit an action potential in the *Response Cell* for $Motif-2$ at pacing period $T = 300$ ms.

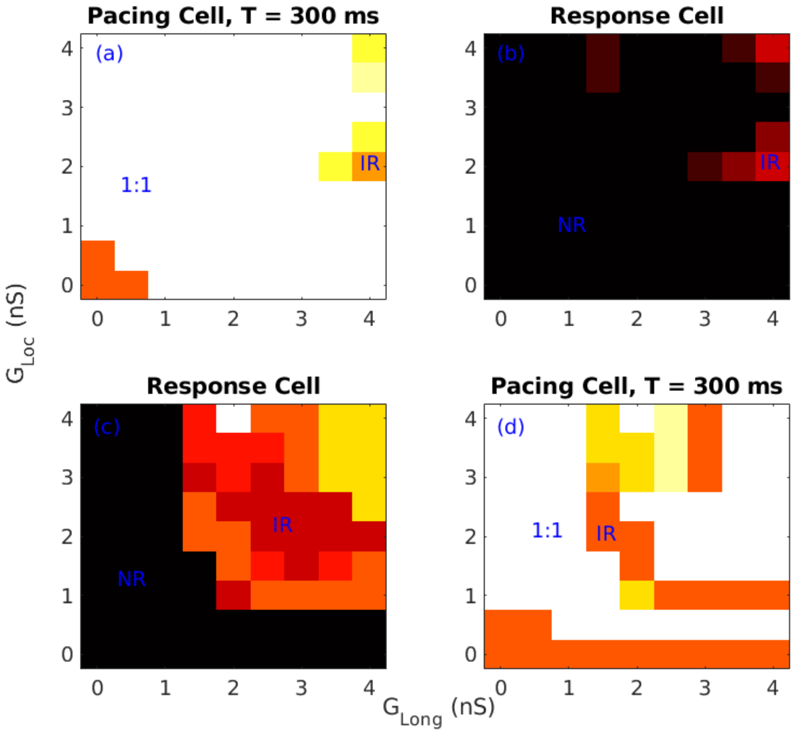

Figure 9: 2-parameter conductance map describing the different dynamical regimes for the *Steep* parameter set, *viz.*, NR, IR and $1:1$, characterised as the fraction of stimuli that elicit an action potential in the *Response Cell* for $Motif-2$ at pacing period $T = 300$ ms.

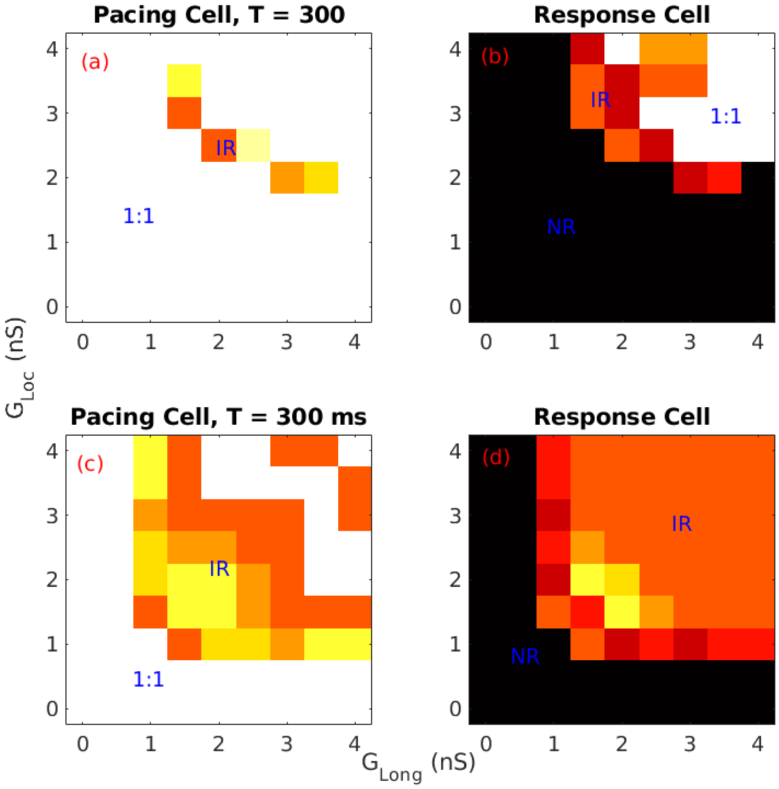

Figure 10: 2-parameter conductance map describing the different dynamical regimes for the *Shallow* parameter, *viz.*, NR, IR and $1:1$, characterised as the fraction of stimuli that elicit an action potential in the *Response Cell* for $Motif-1$ (a,b) and $Motif-3$ (c,d) at pacing period $T = 300$ ms.

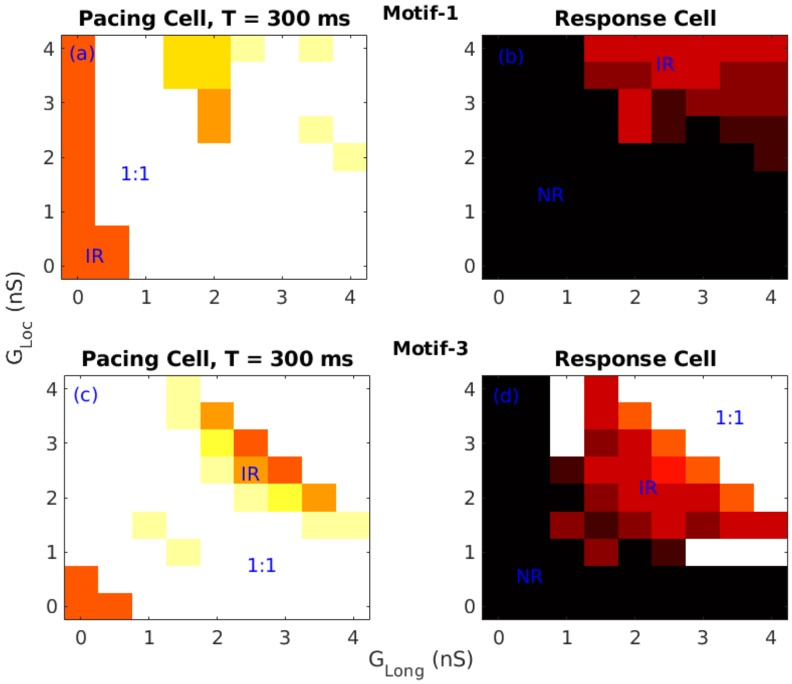

Figure 11: 2-parameter conductance map describing the different dynamical regimes for the *Steep* parameter set, *viz.*, NR, IR and $1:1$, characterised as the fraction of stimuli that elicit an action potential in the *Response Cell* for $Motif-1$ (a,b) and $Motif-3$ (c,d) at pacing period $T = 300$ ms.

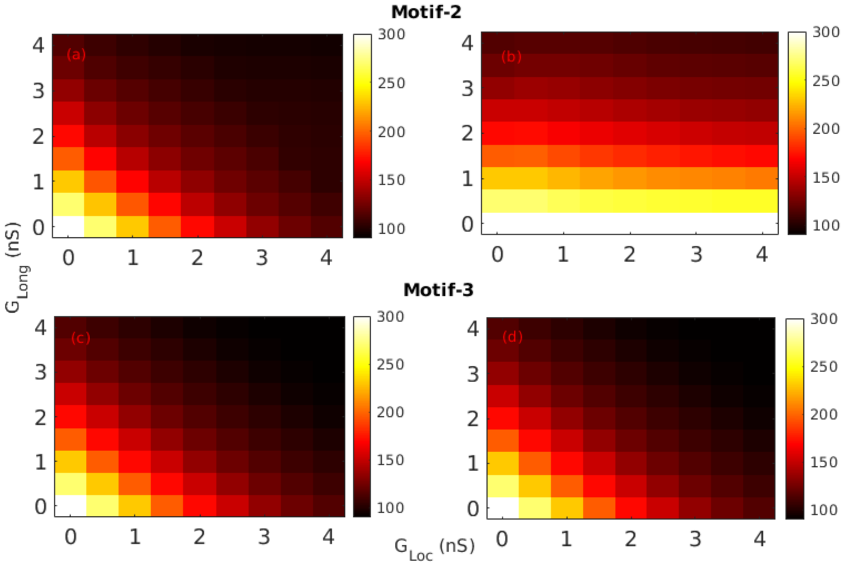

Figure 12: Effect of coupling motifs on APD for *Steep* parameters with $V_{FR} = -49$ mV.

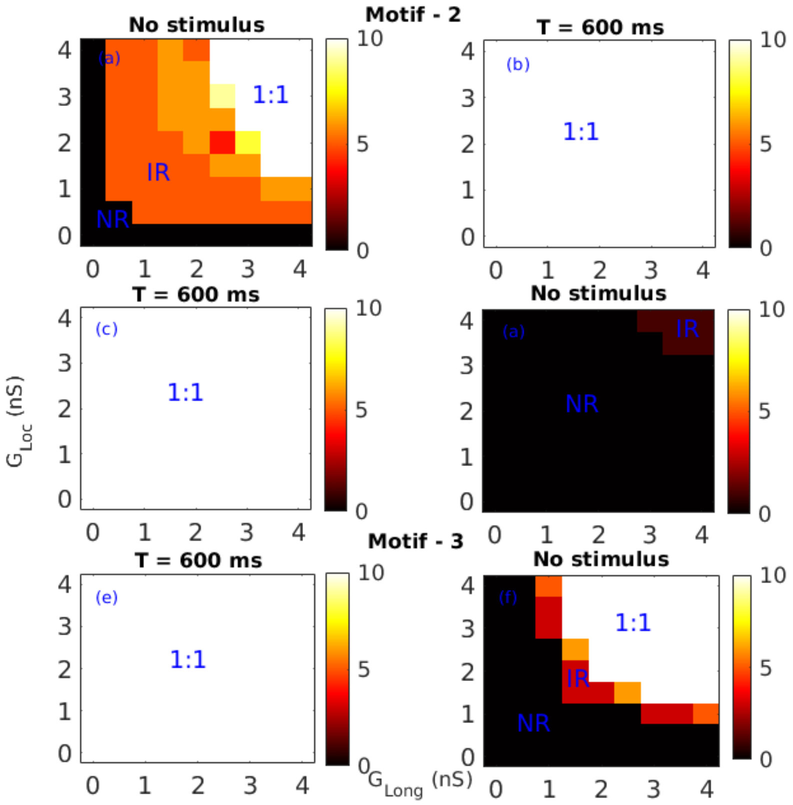

Figure 13: Identifying regimes for *Steep* parameters at $T = 600$ ms that initiate action potentials in non-stimulated cells for $V_{FR} = -49$ mV.

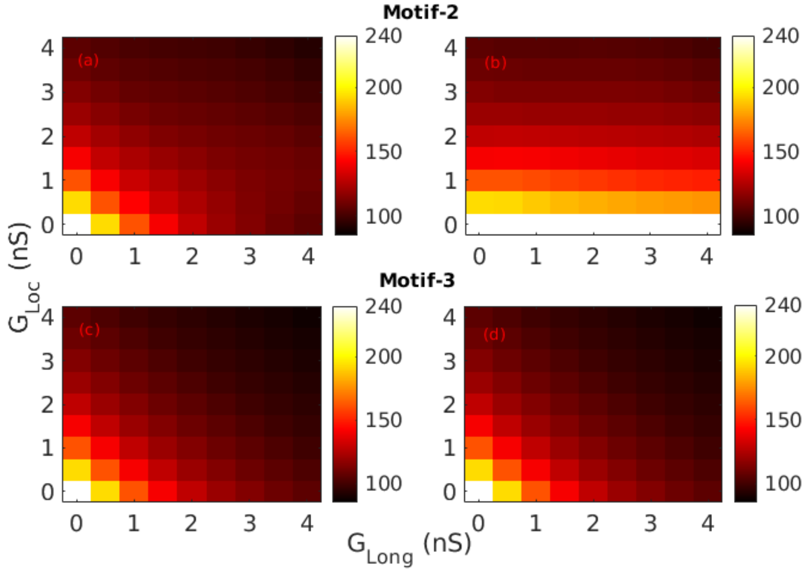

Figure 14: Effect of coupling motifs with *Shallow* cell parameters on APD for $V_{FR} = -49$ mV.

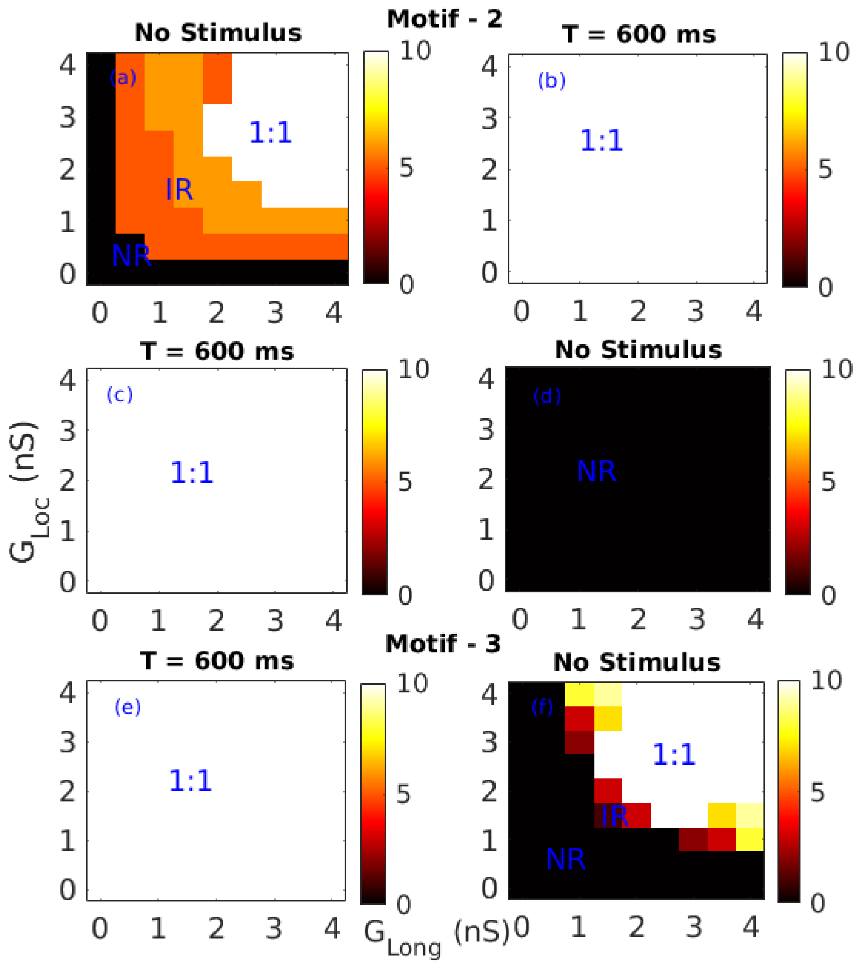

Figure 15: Identifying conductance parameters that initiate action potentials in non-stimulated myocyte with *Shallow* restitution for $V_{FR} = -49$ mV.
